# Recent advances in transfusions in neonates/infants

**DOI:** 10.12688/f1000research.13979.1

**Published:** 2018-05-18

**Authors:** Ruchika Goel, Cassandra D. Josephson

**Affiliations:** 1 Division of Transfusion Medicine, Department of Pathology and Division of Pediatric Hematology/Oncology, Department of Pediatrics, New York Presbyterian Hospital, Weill Cornell Medicine, New York, NY, USA; 2 Departments of Pathology and Pediatrics, Center for Transfusion and Cellular Therapies, Emory University School of Medicine and AFLAC Cancer Center and Blood Disorders Service, Children’s Healthcare of Atlanta, Atlanta, GA, USA

**Keywords:** Neonates, Infants, Premature Infants, Preemies, Transfusion, Red Blood Cells, Plasma, Platelets, Children, Pediatrics

## Abstract

Transfusions of red blood cells (RBCs), platelets, and plasma are critical therapies for infants and neonates (particularly preterm neonates) in the neonatal intensive care unit, who are the most frequently transfused subpopulation across all ages. Although traditionally a significant gap has existed between the blood utilization and the evidence base essential to adequately guide transfusion practices in infants and neonates, pediatric transfusion medicine is evolving from infancy and gradually coming of age. It is entering an exciting era with recognition as an independent discipline, a new and evolving high-quality evidence base for transfusion practices, novel technologies and therapeutics, and national/international collaborative research, educational, and clinical efforts. Triggers and thresholds for red cell transfusion are accumulating evidence with current phase III clinical trials. Ongoing trials and studies of platelet and plasma transfusions in neonates are anticipated to provide high-quality evidence in years to come. This article aims to summarize the most current evidence-based practices regarding blood component therapy in neonates. Data on the use of specific components (RBCs, plasma, and platelets) are provided. We attempt to define thresholds for anemia, thrombocytopenia, and abnormal coagulation profile in neonates to highlight the difficulties in having a specific cutoff value in neonates and preterm infants. Indications for transfusion of specific products, transfusion thresholds, and current practices and guidelines are provided, and possible adverse outcomes and complications are discussed. Finally, the critical research knowledge gaps in these practices as well as ongoing and future research areas are discussed. In an era of personalized medicine, neonatal transfusion decisions guided by a strong evidence base must be the overarching goal, and this underlies all of the strategic initiatives in pediatric and neonatal transfusion research highlighted in this article.

## Background

Blood transfusions can be critical supportive therapy for infants and neonates (particularly preterm neonates) who are frequently transfused ^1, 2^. During their stay in the neonatal intensive care unit (NICU), the majority of extremely low-birth-weight (ELBW) infants receive at least one red blood cell (RBC) transfusion and many end up receiving multiple transfusions. These tiniest of recipients usually receive large volumes of blood products relative to their small size. High-quality research evidence guiding transfusion practice in this patient population has traditionally been lacking, but the pediatric and neonatal transfusion medicine research base has gradually been gaining momentum ^3^. Triggers and thresholds for red cell transfusion are accumulating evidence with current phase III clinical trials. Ongoing trials and studies of platelet and plasma transfusions in infants and neonates are anticipated to provide high-quality evidence in years to come ^4, 5^.

Although transfusions overall are increasingly safe, neonates, especially extremely preterm neonates, are particularly predisposed to transfusion complications, including acute reactions such as transfusion-associated circulatory overload (TACO) and transfusion-associated acute lung injury (TRALI) ^6, 7^, but are also quite vulnerable to short- and longer-term issues resulting from transfusion-transmitted diseases, such as Babesia and Zika virus ^8, 9^. The true incidence of adverse outcomes from transfusions in neonates is not known. These complications tend to be underdiagnosed, especially in neonates with prior respiratory symptoms.

This article aims to summarize the most current evidence-based practices regarding blood component therapy in neonates. Data on specific component use, thresholds, and guidelines are provided. Indications for the transfusion of specific products, including RBCs, platelets, and plasma, and possible adverse outcomes and complications are discussed. Finally, the critical research knowledge gaps in these practices as well as ongoing and future research areas are discussed.

## Red blood cell transfusions in neonates

RBCs are the most commonly transfused blood products in neonates. A majority of ELBW infants require at least one transfusion, and many receive multiple transfusions ^1, 2^. For such a critical therapy in neonates, one would expect the hemoglobin threshold for defining anemia as well as the threshold for transfusion to be well delineated in neonates. However, despite some studies attempting to study this, the lack of an optimal well-defined RBC transfusion threshold for neonates has been consistently recognized as a major gap in our understanding of neonatal transfusion ^4, 5^.

### Defining anemia in neonates

One of the major problems with deciding when to transfuse is the lack of a clearly identified and agreed-upon definition of severe anemia in neonates. Owing to the absence of a universally accepted definition of anemia in neonates necessitating an RBC transfusion and to the wide age-specific reference range of acceptable hematocrit values, treatment thresholds are not standardized. In research settings, various strategies have been employed. Two randomized clinical trials comparing transfusion practices in preterm infants and other prospective studies have used the cutoff hemoglobin level of 8 g/dL or less for defining severe anemia in neonates ^10, 11^. The definition can also be dependent on the outcomes being assessed. For example, preoperative hematocrit has been examined as a continuous variable and a level lower than 40% was found to be the ideal cutoff point (Youden J index) for predicting overall mortality ^12^.

### Red blood cell transfusions in neonates: current practices and evidence

Although there is more convincing randomized controlled trial (RCT) data from adults supporting the use of restrictive transfusion strategies as compared with liberal transfusion strategies, the evidence in neonates is still highly conflicted. In 2005, an RCT by Bell *et al*., studying 100 hospitalized preterm infants with birth weights of 500 to 1,300 g stratified to two levels of hematocrit threshold for RBC transfusion, suggested that, although infants in the liberal transfusion group overall received more RBC transfusions (mean ± standard deviation 5.2 ± 4.5 versus 3.3 ± 2.9), in the restrictive transfusion group, the number of donors to whom the infants were exposed was not significantly different. They additionally reported that there was no major difference between restrictive versus liberal groups in the percentage of infants who “did not get transfusions at all” (12% in the liberal group versus 10% in the restrictive group). Notably, infants in the restrictive group were more likely to have major adverse neurologic events, including (a) intraparenchymal brain hemorrhage, (b) periventricular leukomalacia, and (c) more frequent episodes of apnea (both mild and severe episodes), than the liberal transfusion group. This study raised the possibility that the practice of restrictive transfusions may indeed be harmful to preterm infants ^10^.

The study by Bell *et al*. was followed by the Premature Infants in Need of Transfusion (PINT) study with contrasting results. This RCT included 451 infants with a primary goal to determine whether transfusion of ELBW infants at restrictive versus liberal thresholds had different rates of survival or morbidity at the time of hospital discharge. With a mean birth weight of 770 g and gestational age of 26 weeks, rates of the primary outcome (death before home discharge) were 74.0% in the low-threshold group and 69.7% in the high-threshold group ( *p* = 0.25, risk difference 2.7%, 95% confidence interval [CI] −3.7% to 9.2%). The study revealed that when a higher hemoglobin threshold in ELBW infants was maintained, a greater number of infants received transfusions, with little evidence of benefit ^11^. It is important to note that the trial by Bell *et al*. and the PINT trial were both limited by not studying neurodevelopment beyond hospital discharge. Thus, the interpretation from these studies is restricted to the duration of hospitalization only and not the long-term effects. The shorter-term outcomes studied to date may be a poor proxy for the long-term neurological outcomes. There is limited knowledge on the effects of limiting oxygen delivery to the brain by withholding transfusions, and this remains an important gap in our understanding on the effects of RBC transfusions on neurodevelopment.

Two ongoing and much-awaited definitive phase III transfusion studies in neonates are the Effects of Transfusion Thresholds on Neurocognitive Outcome of Extremely Low Birth Weight Infants (ETTNO) trial and the Transfusion of Prematures (TOP) trial to determine whether higher hemoglobin thresholds for transfusing ELBW infants resulting in higher hemoglobin levels lead to improvement in (1) survival (primary outcome) and (2) rates of neurodevelopmental impairment at 22–26 months of age.

***Age of transfused blood and outcomes in children.*** In children and neonates, as in adults, the age of stored RBCs and adverse outcomes has been a highly debated question. The Age of Red Blood Cells in Premature Infants (ARIPI) RCT showed no difference in outcomes in premature very-low-birth-weight (VLBW) infants requiring a transfusion when fresh RBCs (mean age of 5.1 days) versus standard blood bank practices (mean age of 14.6 days) were followed ^13^. However, there were concerns raised about the generalizability of the ARIPI study. The primary critique was that the mean duration of RBC storage in the standard-of-care group in the ARIPI study was not reflective of the mean storage duration in the US, in which the average storage age was reported to be higher (about 18 days). Furthermore, the hemoglobin thresholds for transfusion were not pre-specified or standardized, allowing for higher hemoglobin levels for each infant, and the storage solution and manufacturing of units in different anticoagulant-preservative solutions were not addressed in the study ^14^. Currently, the Age of Blood in Children in Pediatric Intensive Care Units (ABC PICU) study, examining outcomes comparing transfused RBCs stored for not more than 7 days or standard-issue RBCs (expected mean RBC storage duration of 17–21 days), is awaiting completion. Evidence in older children from the Tissue Oxygenation by Transfusion in Severe Anemia With Lactic Acidosis (TOTAL) randomized clinical trial (290 children 6–60 months old) showed no difference in post-transfusion correction of mean lactate levels between young blood (median age of 8 days) versus older blood (median age of 32 days) ^15^.

*Adverse outcomes from red blood cell transfusions*

RBC transfusions are based on the assumption that the transfusion leads to a direct and immediately effective increase in oxygen delivery to tissues. However, although the correlation with transfused red cell mass and improved oxygenation in neonates has not been proven, RBC transfusions do come with inherent risks for adverse complications in the recipient. Although many of these inherent risks are similar to ones in older children and adults, neonates have some specific adverse associations and outcomes potentially related to RBC transfusions, including the development of necrotizing enterocolitis (NEC), intraventricular hemorrhage (IVH) ^16– 18^, retinopathy of prematurity (ROP) ^19^, and chronic lung disease (CLD) ^20, 21^ as well as mortality. Interpretation of UK analysis of the Serious Hazards of Transfusion (SHOT) scheme data estimated the rates of an adverse outcome to be 18:100,000 red cells issued for children younger than 18 years and 37:100,000 for infants younger than 12 months compared with 13:100,000 for adults ^7^. Thus, infants and neonates are disproportionately more predisposed to adverse effects from RBC transfusions than are other populations.

Meta-analyses/systematic reviews studying these relationships give contrasting results as more and more studies addressing the topics surface. As an example, in 2012, a meta-analysis of 11 retrospective case-control studies and one cohort study showed that recent exposure to transfusion was associated with NEC in neonates. Neonates who developed NEC were at overall higher risk of mortality ^22^. However, a more updated meta-analysis published recently which addresses the same question from 17 observational studies showed no temporal relationship between RBC transfusions and NEC ^23^. This meta-analysis highlighted how the effect size for this relationship differed between matched case-control studies (odds ratio [OR] 1.20, 95% CI 0.58–2.47; *p* = 0.63) and differed from those reported in cohort studies (OR 0.51, 95% CI 0.34–0.75; *p* ≤0.01). This example and contrast in results based on the study type and methodology underscores the need for high-quality prospective evidence in the area.

The important question, however, is whether the adverse effects are from anemia itself or from the transfusions, and methodologically sound prospective studies are required to answer this fundamental question. Patel *et al*., in a prospective study assessing various predictors of adverse outcomes as time-varying co-variates, recently showed that among VLBW infants, severe anemia, instead of RBC transfusions, is associated with an increased risk of NEC, thus proposing the need for further studies to evaluate whether preventing severe anemia is more important than minimizing RBC transfusions ^24^. Likewise, Goobie *et al*. ^12^ recently suggested an association between preoperative anemia and postoperative mortality in neonates ^25^.

Within the transfused group as well, the outcome could vary depending on the hemoglobin transfusion threshold. Keir and Stanworth *et al*. published the most comprehensive review and meta-analysis to date on the harmful effects and associations potentially attributable to RBC transfusions ^26^. Together inclusive of 61 studies (16 randomized trials and 45 non-randomized studies), this meta-analysis revealed no evidence of difference in rates of mortality between restrictive and liberal strategies for transfusion (eight RCTs: risk ratio 1.24, 95% CI 0.89–1.67, heterogeneity = 0%). Similarly, no differences were seen for other outcomes, including NEC, ROP, CLD, or IVH ^26^.

There are no consensus guidelines on how the transfusion practices require specific modification for neonatal use. The standardization of (1) definitions of adverse effects in neonates and (2) associations of RBC transfusion with many of these adverse effects in neonates, through an international consensus of experts, is essential.

## Research gaps and ongoing and future research in red blood cell transfusions in neonates

The majority of research studies thus far are limited by a small sample size; observational, retrospective, or a case-control design; or a lack of evaluation of time-varying exposures to study various outcomes. This underscores the need for prospective studies in which each documentation of anemia, subsequent RBC transfusion, and a specific outcome of interest can be prospectively and systematically evaluated ^27^.

One of the biggest impediments to advancement of neonatal transfusion research is that there are no vein-to-vein databases, registries, or networks that include neonatal RBC transfusion-relevant data with outcome or donor linkage to recipients in sufficient detail ^28^. The dire need of big data applications in transfusion medicine research ^29^ is even more palpable in neonatal transfusion medicine ^28^. This has been identified in scientific proceedings of the National Heart, Lung, and Blood Institute ^30^. However, a few researchers have successfully employed some surgical databases for studying outcomes associated with transfusion and transfusion volumes ^12, 31^. In the absence of these large databases, methodologically sound meta-analyses and systematic reviews can provide key insights and ability to answer some questions ^26^.

## Platelet transfusions in neonates

Estimates of the overall prevalence of neonatal thrombocytopenia vary from 1% to 5% of all neonates and is averaged at about 25% (range of 22–35%) among neonates admitted to NICUs. The most premature of infants have the highest risk of developing thrombocytopenia. Consequently, platelets are the second most commonly transfused blood component in neonates, next to RBCs ^32^.

### Defining thrombocytopenia in infants

Thrombocytopenia in neonates is traditionally defined as a platelet count of less than 150 × 10 ^9^/L and is classified as mild (100–150 × 10 ^9^/L), moderate (50–99 × 10 ^9^/L), or severe (<50 × 10 ^9^/L) ^33^. However, the question is whether there is a standard definition of thrombocytopenia which can be applied to neonates of all gestational ages. The incidence of thrombocytopenia in neonates is extremely variable depending on the gestational age of the studied population. Whereas the overall incidence of thrombocytopenia in newborns at the time of birth is relatively low (less than 1%) ^34^, among NICU patients, the incidence of thrombocytopenia has been reported to be inversely correlated to gestational age. Specifically, among neonates with a birth weight less than 1,000 g, it is estimated to be as high as more than 50% ^35, 36^. In 2009, Wiedmeier *et al*., in a large observational study on neonatal platelet counts, including data on about 47,000 infants (between 22 and 42 weeks of gestational age), suggested a predictable increment in platelet counts since birth with an increase in gestational age, with a mean increase in platelet count of ~2 × 10 ^9^/L per week increase in gestational age ^37^. These findings suggest that there is not one single threshold to define thrombocytopenia in neonates and that different thresholds need to be used to define thrombocytopenia in preterm infants depending on gestational age ^37^.

### Platelet transfusions in neonates: current practices

A clear relationship between low platelet counts and consequential clinical bleeding or major hemorrhage has not been established in neonates ^32^. Several studies have found that a multitude of factors besides the severity of thrombocytopenia predict the bleeding risk in neonates, the degree of prematurity being a predominant factor. Thus, it will be important to develop improved tests to assess primary hemostasis and bleeding risk in neonates. At present, indications, thresholds, and rationale for prophylactic platelet transfusions to correct thrombocytopenia remain controversial and in need of a strong evidence base to guide safe platelet transfusion practices in neonates, especially the most premature infants.

There continues to be widespread heterogeneity in the pretransfusion thresholds for platelet count and overall platelet transfusion practices between various hospitals and countries. In general, moderate thrombocytopenia (50–150 × 10 ^9^/L) is not supposed to be detrimental. In an earlier study of prophylactic platelet transfusion thresholds, over 150 thrombocytopenic neonates less than 33 weeks’ gestation age (weight of 500 to 1,500 g) were randomly assigned to receive platelet transfusions to maintain the platelet count at either greater than 150 × 10 ^9^/L or greater than 50 × 10 ^9^/L in the first week of life. This study suggested that liberal platelet transfusion threshold (150 × 10 ^9^/L) did not protect against IVH ^38^. Another retrospective observational cohort study examined infants with platelet counts of less than 50 × 10 ^9^/L in the NICU, and preterm infants with platelet counts of less than 50 × 10 ^9^/L in whom platelet transfusions were withheld did not suffer from serious hemorrhage, suggesting that lower platelet count thresholds may be safe for otherwise stable infants ^39^. In a multicenter, retrospective cohort study of 972 VLBW infants from six US NICUs, the pretransfusion count was at least 50 × 10 ^9^/L for 653 (65.4%) of 998 transfusions. While platelet transfusion decisions were taken with higher severity of illness, there was neither an association between the severity of thrombocytopenia and the subsequent risk for IVH nor a lower risk of IVH due to platelet transfusions (hazard ratio 0.92, 95% CI 0.49–1.73; *p* = 0.80) ^40^. There is a need for RCT evidence to guide the optimal platelet transfusion decisions in premature neonates.

Studies have also assessed the feasibility of transfusing platelets on the basis of platelet mass (platelet count times mean platelet volume) instead of the platelet count as a transfusion trigger. The use of platelet mass-based NICU transfusion guidelines has been shown in few studies to be feasible as well as safe, as they were associated with fewer platelet transfusions overall and no recognized increase in hemorrhagic problems ^41, 42^.

The major clinical outcome of concern for neonates with severe thrombocytopenia is risk of major bleeding, including intracranial hemorrhage and specifically IVH and periventricular hemorrhage (PVH). Recently, a neonatal bleeding assessment tool (NeoBAT) was proposed aiming for standardized bleeding assessments in this high-risk population ^43^. The NeoBAT score is being applied in prospective studies and trials of neonates which are mentioned subsequently. There are published algorithms which are helpful to direct the diagnosis and specific investigations of different etiologies of thrombocytopenia. Specific indications for platelet transfusions like neonatal alloimmune thrombocytopenia (NAIT) need a high index of suspicion for early diagnosis and prompt attention with specific requirements like antigen-negative platelets.

### Platelet transfusions: current guidelines

The recent guidelines from the British Society of Hematology (BSH) recommend that for stable children younger than 4 months, prophylactic platelet transfusion should be given at a platelet count of less than 30 × 10 ^9^/L and for stable children older than 4 months, prophylactic platelet transfusions should be given at a platelet count of less than 10 × 10 ^9^/L. The guidelines admit that, given the lack of studies in pediatrics, the platelet transfusion indication for critically ill children or those with hematologic and oncologic malignancies who develop severe thrombocytopenia is based on adult study data ^44^.

***Adverse outcomes from platelet transfusions.*** Platelet transfusions come with risks inherent to any blood component transfusion, including clinical errors in administration and incorrect blood component transfused. They also have significant risks of bacterial and septic infections due to storage at room temperature and risks of allergic reaction as well as TACO and TRALI due to the innate plasma component of platelet products.

Although the number of platelet transfusions might be a surrogate marker for level of illness and thus predictive of mortality, Baer *et al*., in a study of 1,600 thrombocytopenic NICU patients, suggested that multiple platelet transfusions may be contributing directly to a small fraction of mortality in neonates ^45^. These relationships remain associations at best and need to be validated in prospectively designed studies.

Data from the UK’s SHOT national hemovigilance scheme against a population-based epidemiological study of transfused patients have indicated that transfusion-associated adverse events are disproportionate in the number of occurrences in the neonatal population as compared with older children and adults. Transfusion reactions are likely highly under-recognized and under-reported in neonates, especially the most premature infants ^7^.

A pathogen-reduced platelet product (INTERCEPT) was approved for use in the US by the US Food and Drug Administration in 2014 and has been used in a small number of neonates worldwide ^46^. Pathogen-reduced platelet products have been used in some European countries for over a decade. The published RCTs comparing the use of pathogen-inactivated platelets with conventional platelets did not specifically enroll neonates. There are few prospective observational studies which have found no evidence of major adverse events in infants (<1 year old). In neonatal patients treated with phototherapy devices with a peak energy wavelength of less than 425 nm, use of the INTERCEPT blood system for the preparation of platelet components is contraindicated owing to a potential for erythema from interaction between ultraviolet light and amotosalen (interceptbloodsystem.com) ^47^. Other pathogen reduction products in the pipeline, such as Mirasol, a riboflavin (vitamin B _2_)-based system, are still in clinical trials. A recent Cochrane review of RCTs comparing the transfusion of pathogen-reduced platelets with standard platelets suggested no evidence of a difference in the incidence of clinically significant bleeding complication or all-cause mortality. The review also suggested that pathogen-reduced platelet transfusions increase the risk of platelet refractoriness and platelet transfusion requirements. Furthermore, evidence on subgroup differences in multiple transfusion trials between the two pathogen-reduced platelet technologies (Intercept and Mirasol) revealed that for all-cause mortality and the interval between platelet transfusions, Intercept was more favorable; however, these studies are not directly applicable to children/neonates ^48^. Longitudinal studies are needed for both products and their use in neonates as well as older children.

### Ongoing and future research in platelet transfusions in neonates

Study of platelet transfusion strategies to prevent or mitigate bleeding in neonatal and adult patients has been long outlined as clinical trial opportunities in transfusion medicine by the National Heart, Lung, and Blood Institute thinktank of experts ^49^. Platelet trigger trials assessing clinically relevant outcomes are now under way in preterm neonates with thrombocytopenia. PlaNeT1 was an observational study of current practice and outcomes of platelet transfusions in neonates. PlaNeT2 ( http://www.planet-2.com) is an ongoing randomized prophylactic threshold trial randomly assigning neonates to a 25 versus 50 × 10 ^9^/L transfusion threshold. The NeoBAT bleeding assessment score is being used by the PlaNeT2 study. Thus far, treatments or research studies with thrombopoietic growth factors have not been undertaken in neonates. Besides studies of the platelet transfusion threshold, studies are needed to characterize the overall platelet function and the global hemostatic profile of neonates, especially preterm infants, as well as the use of thromboelastography (TEG) or thromboelastometry (ROTEM) to monitor hemostatic treatment to better predict their bleeding risk and thus direct prophylactic or therapeutic measures accordingly for IVH/PVH ^50^.

## Plasma transfusion in neonates

Evidence guiding the transfusion of plasma, of all blood products, in neonates is the weakest. Although there are a few prospective multicenter studies exploring plasma use in neonates ^51^, we rely largely on expert opinion to make recommendations for plasma transfusions.

### Definition of “abnormal” coagulation profile in neonates

The neonatal coagulation system matures slowly after birth, and, depending on the levels of various factors at birth, the “normal” reference ranges in neonates and infants are quite different from those in adults. Even within the neonates, the reference ranges vary depending on the gestational age. However, owing to the lack of awareness of the different ranges, transfusion decisions in neonates are unfortunately made on the basis of the adult ranges or “cutoffs” used to define coagulopathy.

### Plasma transfusions in neonates: current practices

A national audit of transfusion practice from the UK indicated that almost 50% of plasma transfusions are given prophylactically to neonates with “abnormal coagulation values” with no evidence of active bleeding, in order to prevent IVH, although there is very low-grade evidence to support the effectiveness of this practice. Likewise, there is widespread use of plasma reported in hospitalized children in the absence of demonstrated efficacy or effectiveness ^52^.

Karam *et al*. and PlasmaTV investigators reported, in an international point prevalence study of plasma transfusions in critically ill children, that about one-third of the plasma transfusions given in a PICU setting are without any evidence of bleeding or an invasive procedure or surgery warranting a plasma transfusion and that only 22% were administered in case of critical bleeding ^53^.

In a follow-up of an *a priori* secondary analysis of a prospective observational study, investigators of the PLASMA TV study reported lower PICU mortality in the solvent detergent versus fresh frozen plasma (FFP)/frozen plasma (OR 0.40, 95% CI 0.16–0.99; *p* = 0.05). Based on these results, the authors suggested that solvent detergent plasma use in critically ill children may be associated with improved survival ^54^. There are no dedicated studies about effectiveness/adverse effects of pathogen-reduced products in term and preterm neonates.

### Plasma transfusions: current guidelines

Current expert opinion supports the “therapeutic” use of plasma either (1) with active bleeding or (2) concurrent to invasive procedures in patients at high risk of bleeding and those recognized to have an abnormal coagulation profile. An abnormal coagulation profile would be identified as the conventional coagulation parameters prothrombin time (PT) or activated partial thromboplastin time (aPTT) being significantly above the normal age and gestation adjusted reference ranges.

The BSH recommends that prophylactic FFP “not” be administered to “non-bleeding” children with minor prolongation of PT/aPTT, including prior to surgery, but it may be considered for surgery to critical sites ^44^. BSH guidelines also advise that plasma not be used as a means for volume replacement or for prevention of bleeding (for example, IVH) in neonates ^44^. 

### Adverse outcomes from plasma transfusions

Plasma use in neonates comes with multiple risks: it could range from a simple allergic reaction secondary to plasma proteins or a febrile non-hemolytic reaction to severe life-threatening reactions, including TRALI, TACO, febrile reactions, and hemolysis, all likely under-recognized and under-reported in neonates.

Although plasma is usually transfused for its hemostatic properties, there have been reports of an association between plasma transfusion and venous thrombotic outcomes in neonatal as well as pediatric patients, with the coagulation factors and platelet-derived microparticles in plasma being considered responsible for the thrombotic effects ^55^. The neonatal study reported a dose-dependent effect with more than five times the odds of thrombosis in neonates who received greater than 50 mL/kg of plasma by day 5 of age. Raffini *et al*., in a study of plasma transfusions in US hospitals, found that the rate of venous thrombosis in children (including neonates) receiving plasma was 10 times greater than the rate for hospital admissions without plasma transfusion ^52^.

### Ongoing and future research of plasma transfusions in neonates

The first necessary step in furthering research and evidence-based data to guide the indications for plasma use in neonates is the establishment of a well-validated and age-specific reference range for coagulation tests in preterm and term neonates and its variability with severity of illness. The ranges must be established for the classic tests, including PT/aPTT/international normalized ratio, as well as for the various global hemostatic assays, including TEG and ROTEM. The next step is having a validated bleeding prediction and assessment battery for neonates to standardize the actual bleeding. Correlation of the prolonged values with actual risk for clinically significant bleeding is another key step, and no correlation has been established so far. Finally, there is a need for RCTs to explore the prophylactic use of plasma in neonates deemed at high risk for bleeding or with abnormal coagulation tests. Hemostatic dysfunction is also prevalent among neonates with moderate-to-severe hypoxic-ischemic encephalopathy and is associated with an increased risk of bleeding and high transfusion burden ^56^.

## Summary of the state of neonatal transfusion medicine

Pediatric transfusion medicine is evolving from infancy and gradually coming of age. It is entering an exciting era with recognition as an independent discipline, a new and evolving high-quality evidence base for transfusion practices, novel technologies and therapeutics, national and international collaborative research, educational and clinical efforts, and enthusiastic and dedicated engagement from not just transfusion experts but also neonatologists, pediatric intensivists, pediatric surgeons, pediatric anesthesiologists, transfusion practitioners, and nursing personnel.

Although harder to study as new transfusion technologies and therapies emerge, it is critically important to specifically study them in neonates, as they are the highest transfused subpopulation in pediatrics. Table 1 provides a list of the aforementioned clinically important issues in neonatal transfusions.

**Table 1.** Summary of clinically important issues in neonatal transfusion.

**Clinically important issues in neonatal red blood cell (RBC) transfusions** 1. Age-specific definition of severe anemia in neonates 2. Restrictive versus liberal transfusion strategies and short-term and long-term effects, especially neurocognitive development 3. The age of stored RBCs and adverse outcomes in premature infants 4. Correlation with the transfused red cell mass and improved oxygenation in neonates 5. Need for established hemovigilance systems to study disproportionate predisposition of infants and neonates to adverse effects from RBC transfusions as compared with other populations 6. Need of big data applications: vein-to-vein databases, registries, or networks to study neonatal RBC transfusions and clinical outcomes
**Clinically important issues in neonatal platelet transfusions** 1. Age-varying thresholds to define thrombocytopenia in neonates 2. Need of established relationship between severity of thrombocytopenia and consequential clinical bleeding or major hemorrhage 3. Validated tools to standardize bleeding assessment in neonates 4. Platelet transfusion thresholds and guidelines specific for stable and critically ill children 5. Safety and efficacy of pathogen-reduced platelet products 6. Characterization of overall platelet function and global hemostatic profile of neonates assessed using thromboelastography or thromboelastometry
**Clinically important issues in neonatal plasma transfusions** 1. Age-specific reference range for coagulation tests in preterm and term neonates to define “normal” and “abnormal” coagulation profile in neonates 2. Efficacy of prophylactic plasma transfusions to “non-bleeding” neonates to correct “abnormal coagulation values” 3. Plasma transfusion guidelines in bleeding children or those undergoing invasive surgeries 4. Validated bleeding prediction and assessment battery for neonates to standardize the actual bleeding and guide plasma transfusion therapy 5. Adverse outcomes from plasma transfusions in neonates 6. Hemostatic dysfunction among neonates with specific clinical scenarios like moderate-to-severe hypoxic-ischemic encephalopathy and role of plasma transfusions 7. Characterization of the global hemostatic profile of neonates assessed using thromboelastography or thromboelastometry and role of plasma transfusions

We continue to rely on expert opinion to make many transfusion-related recommendations in infants and neonates, but with an increased focus on research in neonatal transfusion practice, this is anticipated to change and improve with time.
